# Irinotecan is active in chemonaive patients with metastatic gastric cancer: a phase II multicentric trial

**DOI:** 10.1038/sj.bjc.6601226

**Published:** 2003-09-09

**Authors:** C-H Köhne, R Catane, B Klein, M Ducreux, P Thuss-Patience, N Niederle, M Gips, P Preusser, A Knuth, M Clemens, R Bugat, I Figer, A Shani, B Fages, D Di Betta, C Jacques, H J Wilke

**Affiliations:** 1 Robert Rossle Klinik, Charité University Hospital, Berlin, Germany; 2 Shaare Zedek Medical centre, Jerusalem, Israel; 3 Rabin Medical Centre, Oncology, Golda Campus, Petach-Tikva, Israel; 4 Institut Gustave Roussy, Villejuif, France; 5 Klinikum Leverkusen, Leverkusen, Germany; 6 Hadassah Ein Karem Medical Centre, Jerusalem, Israel; 7 Chirurgische Onkologie, Department of General Surgery, Muenster University Hospital, Germany; 8 II Medizinische Klinik Krankenhaus Nordwest, Frankfurt, Germany; 9 Krankenanstalt Mutterhaus der Borromaerinnen, Trier, Germany; 10 Institut Claudius Regaud, Toulouse, France; 11 Beilinson Medical center, Petach-Tivka, Israel; 12 Kaplan Medical Center, Rehouot Netanya, Israel; 13 Aventis, Antony, France; 14 Kliniken Essen Mitte, Essen, Germany

**Keywords:** phase II, irinotecan, metastatic gastric cancer

## Abstract

To assess the response rate and the tolerance of irinotecan as first-line therapy, 40 patients with metastatic gastric cancer received irinotecan 350 mg m^−2^ every 3 weeks administered as a 30 min infusion. Among the 35 patients evaluable for response, two complete and five partial responses were recorded (response rate: 20.0% (95% CI:8.4–36.9%)). In total, 16 patients achieved stable disease and 12 progressive disease. In all, 66 percent of the patients benefited from tumour growth control. The median time to progression was 3.0 months (95% CI: 2.3–4.4%). The median overall survival was 7.1 months (95% CI: 5.2–9.0%). The probability of being alive at 6 months and 9 months was 61.0 and 32.4%, respectively. The median number of cycles per patient was 3 (range 1–14), and the relative dose intensity was 0.98. The most common grade 3–4 toxicities by patients were diarrhoea 20%, asthenia 10%, nausea 7.5%, vomiting 5.0%, abdominal pain 5%, neutropenia 38.5%, leucopenia 28.2%, anaemia 12.8% and thrombocytopenia 5.1%. Febrile neutropenia occurred in 12.5% of patients. These findings indicate that irinotecan is active and well tolerated in patients with metastatic gastric adenocarcinoma and warrants further evaluation in this clinical setting.

Gastric carcinoma is a highly aggressive disease with an exceedingly poor prognosis for patients with advanced stage disease. It is the fifth most common malignancy in the European Union and is the fourth most common cause of cancer death after lung cancer for men, breast cancer for women and colorectal cancers for both. While a decreased incidence of gastric adenocarcinoma has been observed for several decades in Western countries, this has been accompanied by an increase in the incidence of tumours of the gastro-oesophageal junction and a simultaneous shift towards poorly differentiated adenocarcinomas (Blot *et al*, 1991). Most gastric tumours are diagnosed at an advanced stage, and surgery, even if performed with curative intent, gives disappointing results with an overall 5-year survival of 10% (Maruyama *et al*, 1987; Wanebo *et al*, 1993; Bonenkamp *et al*, 1999). The causes of surgical failure are a combination of local recurrence and distant metastases.

Chemotherapy is able to prolong survival relative to best supportive care (Murad *et al*, 1993; Pyrhonen *et al*, 1995; Glimelius *et al*, 1997). Response rates of 5%–21% have been reported for single-agent chemotherapy, with 5-fluorouracil (5-FU), in chemonaive patients (Wils and Bleiberg, 1989; Loehrer *et al*, 1994). Other agents, when used as monotherapy, have produced response rates as follows: paclitaxel 20% (Ajani *et al*, 1998), adriamycin 24% (O'Connell, 1985), docetaxel 25% (Sulkes *et al*, 1994), cisplatin 25% (O'Connell, 1985), UFT 28% (Takiuchi and Ajani, 1998) and mitomycin C 30% (Comis and Carter, 1974).

At present, combination chemotherapy is the standard treatment for advanced disease. Several regimens have been used (FAM, FAMTX, ECF, EAP, ELF, FP), but those containing cisplatinum in combination with infusional 5-FU are the most widely accepted. Unfortunately, any improvements in response rate only translate into a small benefit in terms of overall survival, and median durations of survival that are still between 6 and 10 months (Köhne *et al*, 2000). Thus, any active agent with a new mechanism of action deserves further investigation.

Irinotecan (CPT-11, Campto®) is an S-phase-specific, semisynthetic derivative of camptothecin which interferes with DNA replication and cell division through its potent interaction with the enzyme topoisomerase I. A statistically significant survival advantage has been shown for irinotecan as a single agent, in patients with colorectal cancer who had failed on prior 5-FU-based therapy (Cunningham *et al*, 1998; Rougier *et al*, 1998), and for irinotecan in combination with infusional and bolus 5-FU/folinic acid regimens, first line in the treatment of patients with metastatic colorectal cancer (Douillard *et al*, 2000; Saltz *et al*, 2000). In metastatic gastric cancer, the activity of irinotecan as a single agent has been reported by Futatsuki *et al*. (1994) in 60 evaluable patients treated at a dose of 100 mg m^−2^ every week or 150 mg m^−2^ every 2 weeks. The response rate in this trial was 23%. In two phase II studies (Wakui and Taguchi, 1992; Lin and Hecht, 2000) using different schedules of irinotecan: 100 mg m^−2^ once per week, 150 mg m^−2^ every 2 weeks, 200 mg m^−2^ every 3 weeks, 50 mg m^−2^ day for 3 days bi-weekly (Wakui and Taguchi, 1992) and 125 mg m^−2^ weekly for 4 weeks followed by 2 weeks rest (Lin and Hecht, 2000), the response rates were 12 and 17%, respectively. The present study investigated the response rate, survival and toxicity profile of irinotecan, 350 mg m^−2^, as a single agent, administered every 3 weeks, in chemotherapy naive patients with metastatic gastric adenocarcinomas.

## PATIENTS AND METHODS

### Patient selection

Patients with a histologically confirmed diagnosis of metastatic gastric adenocarcinoma with at least one bidimensionally measurable lesion were eligible for recruitment into the study. Other eligibility criteria were: age between 18 and 70 years old, a WHO performance status ⩽2, no prior chemotherapy (neither palliative nor (neo)adjuvant), at least 3 weeks from surgery and 6 weeks from radiotherapy, adequate haematopoietic (haemoglobin>10 g dl^−1^, absolute neutrophil count (ANC)>2.0 × 10^9^ l^−1^, platelets>150 × 10^9^ l^−1^), renal (adequate serum creatinine ⩽1.25 × upper normal limit (UNL)) and hepatic (total serum bilirubin ⩽1.25 × UNL, AST and ALT⩽3 × UNL or in case of liver metastasis, total bilirubin⩽1. 5 UNL, AST and ALT⩽5 × UNL) function. Exclusion criteria consisted of locally advanced disease without distant metastases, tumour types other than adenocarcinoma (leiomyosarcoma or lymphoma), the presence of a central nervous system (CNS) metastasis, pregnancy or lactation, prior or current history of chronic diarrhoea, bowel obstruction, subacute bowel obstruction, Crohn's disease or ulcerative colitis, other serious illnessess or medical conditions (e.g. current infection; active disseminated intravascular coagulation), past or concurrent history of neoplasm other than gastric carcinoma, except for curatively treated non melanoma skin cancer or *in situ* carcinoma of the cervix. All patients provided written informed consent prior to entry into the study. The study was approved by the Ethics Committees of all the participating institutions.

### Treatment

Treatment consisted of irinotecan i.v. 350 mg m^−2^ administered day 1 every 3 weeks as a 30 min infusion. Two subsequent dose reductions (to 300 mg m^−2^ and if needed to 250 mg m^−2^) were planned in the case of either haematological toxicity as defined by a nadir with grade 4 neutropenia, grade 3–4 neutropenia with concomitant fever ⩾38.1°C, or in the case of severe diarrhoea (grade 3–4 NCI-CTC) requiring rehydration. Treatment was delayed by 1–2 weeks if the ANC was <1.5 × 10^9^ l^−1^ or the platelet count was <150 × 10^9^ l^−1^ on the day of treatment. Treatment was to be given until progressive disease (PD), unacceptable toxicity or withdrawal of patient consent. Patients with stable disease (SD) and no symptomatic improvement at the first assessment were allowed to switch to a salvage treatment. Premedication with antiemetics as well as treatment with atropine in the case of previous severe cholinergic syndrome was required.

### Efficacy assessment

Response rate was the primary end point of this study. A baseline tumour evaluation was required within the 15 days prior to registration. Tumour assessments while on study were performed every two cycles. WHO guidelines were used to assess tumour response. All responses were confirmed at least 28 days later.

Radiological documentation for all patients was reviewed by a panel of external experts. To be evaluable for response, a patient had to have at least one bidimensionally measurable lesion, to have received at least two cycles of treatment and have been assessed with the same method of measurement as at baseline. Patients who progressed before the first assessment at cycle 2 were considered to have PD. Subjective patient improvement (yes/no) was assessed by the investigator at each cycle. Survival was defined as the interval from the first day of treatment to the date of death. The time to progression (TTP) was the interval from the first day of treatment to the date of progression.

### Safety assessments

Patient history, baseline signs and symptoms, full blood count and chemistry (alkaline phosphatase, AST, ALT, total and direct bilirubin, electrolytes and creatinine) were performed within the 8 days prior to registration and thereafter prior to the commencement of each cycle. A blood count was performed weekly. The NCI common toxicity criteria (NCI-CTC) scale was used in grading the adverse events during the study. Adverse events, signs and symptoms were determined at the end of each cycle. All adverse events occurring within 30 days of the last irinotecan infusion were followed. Adverse events related to irinotecan that were still ongoing 30 days from the time of the last irinotecan infusion were also followed until resolution.

### Statistical methodology

The purpose of this phase II trial was to investigate the efficacy of irinotecan as a single agent in 30 evaluable patients with advanced gastric carcinoma who had received no prior chemotherapy. A two stage modified Fleming design (Fleming, 1982) was used. A total of 15 patients were planned at each stage except in case of early termination after the first stage. If no responses were observed in the first stage, the trial was to be stopped. If at least one response was observed, at least 15 additional evaluable patients were to be accrued. If less than four responses were observed in the total population of 30 patients, the final conclusion was to be that the activity of irinotecan was disappointing. If at least four responses were observed, then the activity of irinotecan as demonstrated in this study would have been sufficiently promising to warrant further development. The procedure tests, the null hypothesis (H_0_) as formulated for this study that the true response rate is ⩽5% *vs* the alternative hypothesis (H_A_) that the true response rate is ⩾25%, with a significance level of 6% and a power of 96%. Survival curves were estimated by the Kaplan–Meier method. The confidence interval of the median was calculated using the Brookmeyer and Crowley method (Brookmeyer and Crowley, 1982).

The dose intensity administered per week was calculated dividing the actual delivered dose by the length of the cycle (i.e. 3 weeks or longer in case of treatment delays). The relative dose intensity was calculated by dividing the actual dose intensity by the planned dose intensity.

## RESULTS

### Patient characteristics

In all, 40 patients were accrued between 18/03/96 and 04/06/98 into this multicentre, nonrandomised, open-label phase II study. One patient with an oesophageal adenocarcinoma was ineligible. Four patients (10%) were not evaluable for response: one patient died from cardiac congestive failure, another patient discontinued before the first assessment due to febrile neutropenia and diarrhoea and tumour assessments were not properly performed in two other patients. In total, 35 patients (87.5%) were evaluable for response. The patient characteristics for all patients are listed in Table 1

**Table 1:** Treated patient characteristics

	***N*=40**	**%**
Sex: male/female	28/12	70/30
Median age (range)	58.0 (36–74)	—
Median weight loss during last 3 months (range)	5% (0.0–16.0)	—
Performance status (0–1–2)	13–23–4	33/58/10
*Gastric adenocarcinoma*	39	97.5
Undifferentiated	21	52.5
Well differentiated	17	42.5
Not available	2	5.0
*Status*		
Metastatic with primary tumour	25	62.5
Metastatic without primary tumour	15	37.5
*Number of organs involved*		
1–2	34	85.0
⩾3	6	15.0
*Sites of disease*		
Lymph nodes	24	60.0
Liver	22	55.0
Lung	8	20.0
Others	14	35.0
*Previous treatment*		
Prior radiotherapy (yes/no)	1/39	2.5/97.5
Prior surgery (yes/no)	22/18	55.0/45.0
Intent of surgery (curative/palliative)	8/14	20.0/35.0
Median time (months) between surgery and study entry	4.0 (0.3–65.1)	
*Signs and symptoms*		
No symptom at baseline	10	25.0
Symptoms at baseline	30	75.0
At least one tumour-related symptom	28	70.0

. The gender ratio (male/female) was 28/12 (70/30%). The majority, 36 patients (90%), had a good performance status (0–1). The median weight loss experienced was 5.0% (range: 0.0–16.0). In all, 21 patients (52%) had undifferentiated tumours. A total of 22 (55%) patients (21 evaluable patients) had had prior surgery. In only 20% was it conducted with curative intent, 34 patients (85%) had one or two organs involved. A total of 30 patients (75%) had symptoms at baseline and of these, 28 (70%) had at least one tumour-related symptom.

### Drug delivery

In total, 40 patients received 163 cycles of irinotecan. The median number of cycles per patient was 3 (range 1–14). The median dose intensity was 114 mg m^−2^ week^−1^, with a median relative dose intensity of 0.98. Of the 163 cycles 124 (76%) were administered at the full dose; only 25% (10) of the patients, and 8.6% (14) of the cycles were delayed, mostly for nondrug-related reasons. The dose of irinotecan was reduced in 23% (9) of the patients, but in only 6.1% (10) of the cycles. The reasons for dose reduction were haematological toxicity for two cycles, non–haematological toxicity for five cycles and both for three cycles. Nine cycles were delayed for 4–7 days and 5 cycles were delayed for more than 7 days. In total, 26 patients had neither a dose reduction nor a dose delay. These data confirm the feasibility of both the dose and the schedule.

### Efficacy

Out of 35 evaluable patients, there were two confirmed complete responses (CRs) and five confirmed partial responses (PRs). In total, 16 patients had stable disease (SD) and 12 PD. The overall response rate (Table 2

**Table 2:** Responses and response rate: patients evaluable for response

**Response rate**	***N*=35**	**%**
Complete response	2	5.7
Partial response	5	14.3
No change	16	45.7
Progressive disease	12	34.3
Response rate (95%CI)	20.0 (8.4–36.9)	

), for the evaluable patient population, was 20.0% (95% CI: 8.4-36.9%), and 66% of the patients benefited from tumour growth control. Of the 40 treated patients (intention-to-treat (ITT) population), the overall response rate was 17.5% (95% CI: 7.3–32.8%). The median time to first response was 2.9 months. Two responses were observed at cycle 2, two responses (including one CR) at cycle 4, one response (a CR) at cycle 8 and two responses at cycle 9. For the complete responders, one patient presented with multiple liver lesions at baseline, and the other with a coeliac mass (39 mm × 28 mm). Responses were observed at all sites, although it should be mentioned that one patient categorised as having SD experienced a CR for all sites except the skin, and one patient categorised as PR experienced CR at the metastatic site although the primary tumour remained. Among the seven responding patients, the number of cycles of irinotecan administered varied between six and 13 cycles. Of note, 16 patients had a ‘best response’ of no change (SD), during their treatment with irinotecan. The median duration of response and disease stabilisation was 4.4 months. In all, 17 (48.6%) patients had at least one symptomatic improvement during treatment, which were observed from the first (14 patients) and second (three patients) cycles. Among them, five (14.3%) patients had a PR, 11 (31.4%) patients had SD and one patient had PD. At the first tumour assessment (at the end of cycle 2), a total of 12 (37.5%) patients out of the 32 patients receiving a second cycle, reported a symptomatic improvement.

**Figure 1:**
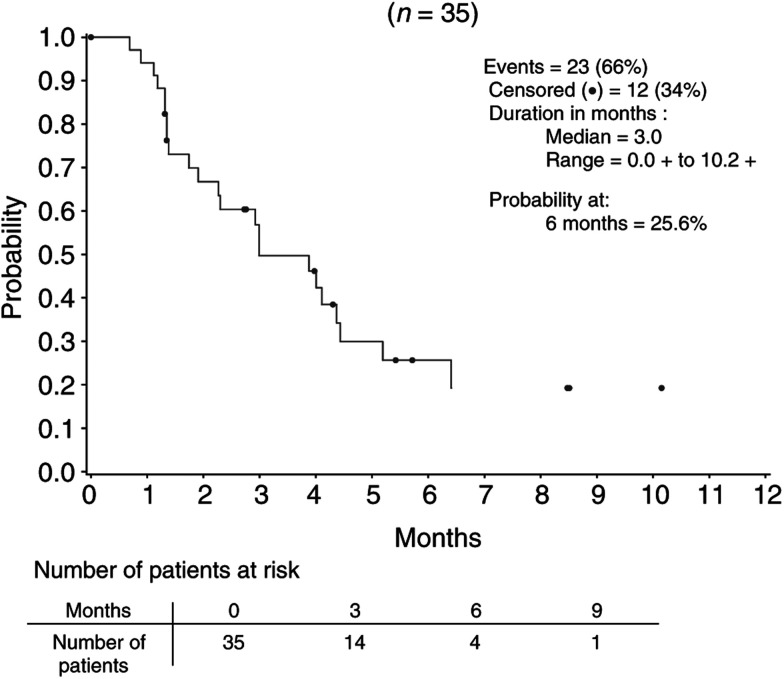
Time to progression in patients evaluable for response.

**Figure 2:**
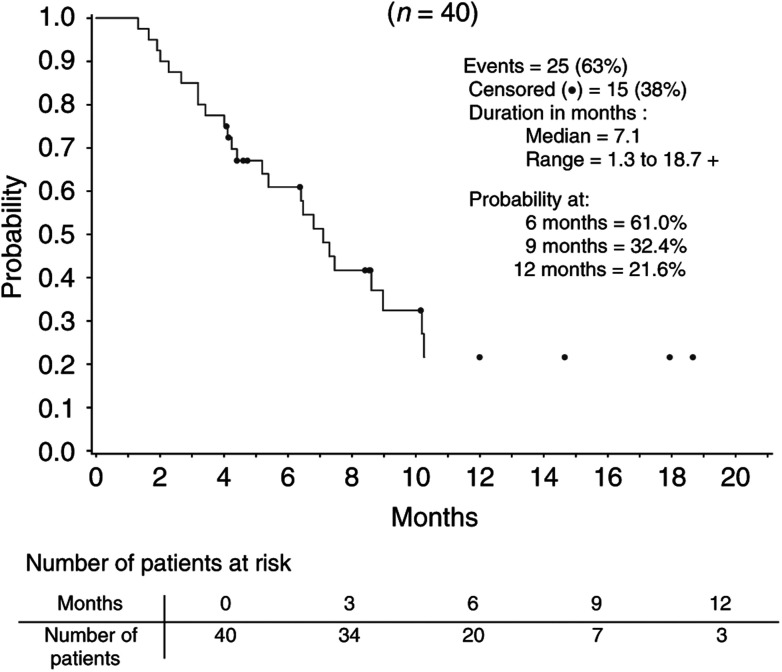
Survival in treated patients (ITT population).

The median TTP (Figure 1

) was 3.0 months (95% CI: 2.3–4.4%). The probability of being free from progression at 6 months was 26%. With a median follow-up of 9.6 months, the median survival time (Figure 2

) was 7.1 months (95% CI: 5.2–9.0%). The probability of being alive at 6 and 9 months was 61.0 and 32.4%, respectively. In all, 19 patients received further chemotherapy after irinotecan discontinuation. For 10 of these patients, this consisted of cisplatin and 5-FU. All but one of these 19 patients had documented PD. The remaining patient went ‘off study’ after the first cycle due to an adverse event, and received further treatment 3 weeks later.

### Tolerance

In general, the safety profile of irinotecan was good and was found to be consistent with the safety profile of the drug in other indications (Table 3

**Table 3:** Grade 3–4 adverse events classified according to NCI-CTC

	**Patients (*N*=40) *N* (%)**			**Cycles (163) *n* (%)**		
**Grade 3–4 NCI/CTC adverse events**	**Grade 3**	**Grade 4**	**3+4**	**Grade 3**	**Grade 4**	**3+4**
Anaemia^a^	4	1	5 (12.8)	5	1	6 (3.7)
Leucopenia^a^	5	6	11 (28.2)	11	7	18 (11.2)
Neutropenia^a^	7	8	15 (38.5)	13	13	26 (16.5)
Thrombocytopenia^a^	—	2	2 (5.1)	—	2	2 (1.2)
Febrile neutropenia	5	—	5 (12.5)	6	—	6 (3.7)
Infection with concomitant gr3–4 neutropenia	1	—	1 (2.5)	1	—	1 (0.6)
Diarrhoea	5	3	8 (20.0)	6	3	9 (5.5)
Asthenia	4	—	4 (10.0)	4	—	4 (2.5)
Nausea	3	—	3 (7.5)	3	—	3 (1.8)
Vomiting	2	—	2 (5.0)	2	—	2 (1.2)
Abdominal pain	1	1	2 (5.0)	1	1	2 (1.2)

a *N*=39 evaluable patients (with at least one blood count).

*n*=161 evaluable cycles (with at least one blood count) except for neutropenia (*n*=158 cycles).

). Neutropenia grades 3–4 was the most common haematological side effect and was experienced by 15 patients (38.5%) during 26 (16.5%) cycles. Four patients received granulocyte-colony stimulating factor (G-CSF). No prophylactic G-CSF was administered during the course of this study. Febrile neutropenia occurred in five patients (12.5%) during six cycles (3.7%). Infection with concomitant grade 3–4 neutropenia was observed in one patient during one cycle. Diarrhoea was commonly reported (77.5% (31) of the patients and in 44.2% (72) of cycles). Overall, eight patients (20%) experienced severe diarrhoea during 5.5% of cycles, and four of these required hospitalisation. Nausea was frequent (62.5% (25) of patients, 37.4% (61) of cycles), but was rarely severe (7.5% (3) of patients and 1.8% (3) of cycles). Asthenia occurred in 20.0% (8) of the patients and 6.1% (10) of the cycles, but was also rarely severe 10.0% (4) of patients and 2.5% (4) of cycles. Grade 1–2 alopoecia was common (60% (24) of patients: 15% (6) grade 1 and 45% (18) grade 2). Cholinergic syndrome was observed in 50% (20) of patients and in 21.5% (35) of cycles, but was never severe. One patient (69 years old) died while on study: the patient experienced febrile neutropenia, grade 1 diarrhoea and grade 1 dehydration and hypocaliemia during cycle 2. Fluid substitution was followed by deterioration of the patient's general condition and the onset of a lethal congestive cardiac failure. This death was considered to be drug related by the investigator, as the patient had no relevant previous history of cardiac disease.

## DISCUSSION

This phase II study is the first reported European trial of irinotecan for the treatment of metastatic gastric adenocarcinoma.

With an objective response rate of 20.0% including two CRs, this study confirms that irinotecan is active in the treatment of this dismal disease. The response rate deserves several comments. Firstly, CRs are uncommon with single-agent treatment. The occurrence of these CRs not only confirms the antitumour activity of irinotecan, but ranks irinotecan among the most active agents in this indication. Secondly, it is worth mentioning that due to the activity of the drug, the responding patients were able to receive a long period of treatment that was associated with a significant clinical benefit. Thirdly, as already observed with irinotecan for colorectal cancer, responses may occur at any time during treatment, and justifies the continuation of the treatment in patients experiencing clinical benefit from irinotecan.

A total of 46% of the evaluable patients had SD as their best response, with a median duration of treatment of three cycles. This corresponded to the number of patients (37.5%) who had experienced a subjective improvement of their status at cycle 2.

With a median follow-up for survival of 9.6 months, the median survival has been reached at 7.1 months, with a probability of being alive at 9 months of 32.4%. However, this promising survival may also be linked to the fact that 47.5% of the patients went on to receive further active chemotherapy (principally of cisplatinum/5-FU). Responses to the combination cisplatinum/5-FU have been documented previously after irinotecan failure, but they were of short duration (Thuss-Patience *et al*, 2002). Nevertheless, these results show that other cytotoxics commonly used in the treatment of gastric cancer are not cross-resistant with irinotecan. This had already been suggested by Japanese data, where patients treated with chemotherapy second line experienced some activity (Futatsuki *et al*, 1994). However, currently there is no proof that second-line chemotherapy treatment significantly improves survival in patients with gastric cancer.

As mentioned earlier, several factors including the high median dose intensity, the high percentage of cycles administered at the full dose, the low proportion of delayed cycles (mostly for nondrug-related reasons) as well as the low proportion of reduced cycles (mostly for nonhaematological reasons), confirm the feasibility of the dose and the schedule. Thus, irinotecan as a single agent was very well tolerated in these patients. The haematological and nonhaematological safety profiles observed for the present cohort of patients with metastatic gastric cancer compare favourably with the experience of irinotecan in patients with advanced CRC. The incidence of severe diarrhoea (20.0%) is comparable to that observed in phase II–III trials using high-dose loperamide (Cunningham *et al*, 2000). The safety results of this study were also similar to those obtained previously for irinotecan in CRC patients. However, Egner *et al* (1999) reported the excessive toxicity of irinotecan at a dose of 320 mg m^−2^, firstline, in nine patients with advanced gastric or gastro-oesophageal junction adenocarcinomas. Six of the nine patients in this study were hospitalised for grade 4 toxicity (67%), prompting the authors to conclude that the irinotecan dose of 350 mg m^−2^, while tolerable in patients with CRC was too toxic in patients with gastric cancer. The results of the current phase II study do not support this conclusion, since the proportion of toxicity-related hospitalisations in this study was 25%, or 10 patients out of 40.

In conclusion, the activity of irinotecan in the treatment of metastatic gastric cancer is confirmed. The present phase II trial ranks irinotecan as one of the most active agents in this indication based on the presence of confirmed CRs. Irinotecan is also well tolerated, as demonstrated by the excellent relative dose intensity. Based on these promising results, randomised phase II and III trials of combination regimens including irinotecan are ongoing (Pozzo *et al*, 2001) and will help to define the role of this drug further in the treatment of gastric cancer.
